# The Endoscopic Optimization for Obesity in Patients Undergoing Abdominal Wall Reconstruction

**DOI:** 10.3389/jaws.2025.15383

**Published:** 2025-10-24

**Authors:** Victoria L. Walker, Samantha W. Kerr, Grace N. LaFleur, B. Todd Heniford, Sullivan A. Ayuso

**Affiliations:** ^1^ Department of Surgery, Baptist Health Sciences University, Memphis, TN, United States; ^2^ Division of Gastrointestinal and Minimally Invasive Surgery, Department of Surgery, Carolinas Medical Center, Charlotte, NC, United States; ^3^ College of Osteopathic Medicine, Baptist Health Sciences University, Memphis, TN, United States; ^4^ Division of Gastrointestinal and General Surgery, Department of Surgery, Endeavor Health, Evanston, IL, United States; ^5^ Division of Elective General Surgery, Department of Surgery and Perioperative Care, Dell Medical School, University of Texas at Austin, Austin, TX, United States

**Keywords:** ventral hernia, abdominal wall reconstruction, prehabilitation, obesity, endoscopic sleeve gastroplasty

## Introduction

Obesity significantly increases the risk of complications following abdominal wall reconstruction (AWR), including surgical site infections (SSIs), wound dehiscence, and hernia recurrence rates of up to 35%–40% [1–3]. A BMI threshold of BMI ≥35 kg/m^2^ has typically been identified as a threshold at which these risks rise substantially; however, prior data has shown that there is an increase starting at lower BMIs [4, 5]. With global obesity rates continuing to rise, hernia surgeons are increasingly tasked with managing high-risk patients whose comorbidities and functional limitations require careful optimization prior to surgery when possible [6]. AWR is a benign procedure performed to restore function and improve quality of life rather. This distinction underscores the importance of maximizing perioperative outcomes, as the central goal is to achieve durable repair and enhance functionality. Despite this, there is currently no standardized, stepwise approach to prehabilitation that incorporates endoscopic bariatric therapies. This gap in clinical practice highlights an opportunity to integrate endoscopic weight-loss strategies, specifically endoscopic sleeve gastroplasty (ESG), into the preoperative pathway for patients undergoing AWR. This manuscript explores the rationale, benefits, limitations, and practical considerations of ESG as a potential prehabilitation strategy to improve outcomes in this high-risk population.

## Rationale for Weight Loss Prior to Abdominal Wall Reconstruction

Weight loss prior to AWR improves both technical and clinical outcomes [7]. Reducing visceral and subcutaneous adiposity can facilitate fascial approximation, reduce tension at the repair site, and lower the risk of postoperative complications [8]. Enhancing a patient’s functional capacity before surgery can also contribute to optimal outcomes by improving cardiopulmonary function, glycemic control, and overall patient resilience, factors that collectively reduce surgical risk and improve the overall health of the patient [5, 9]. In our practices at University of Texas Health Austin and Endeavor Health, implementation of a ketogenic diet supplemented by daily aerobic exercise has been effective in optimizing patients for surgery and resulted in sustained improvements in health following surgery [10]. While we do not have a specific cut point for body mass index (BMI) prior to open AWR, in general, we encourage weight loss in all overweight and obese patients as each unit of BMI is associated with a significant reduction in wound complications [3]. In patients with large hernias, weight loss has the important impact of facilitating fascial closure by reducing hernia and intra-abdominal volume, and it also reduces tension on the closed wound. Previous studies demonstrate that lack of fascial closure increases recurrence rates by 7-fold and that increasing obesity directly correlates with elevated intra-abdominal pressure [11, 12]. Traditionally, these lifestyle modifications (e.g., ketogenic diet and daily aerobic exercise) have been the cornerstone of perioperative weight management, whereas surgical or endoscopic weight loss interventions were typically reserved for patients with more severe obesity or those without urgent indications for hernia repair. The purpose of this opinion piece is to advocate for the inclusion of ESG as a complementary strategy within the existing perioperative optimization paradigm. In particular, this may be a valuable emerging technology to achieve a longer-term effect in comparison to lifestyle modifications alone.

## Primary Endobariatric Therapies and Clinical Benefits

Two principal endobariatric options for weight loss include intragastric balloon (IGB) therapy and endoscopic sleeve gastroplasty (ESG). IGB involves the endoscopic placement of a saline-filled or air-filled balloon in the stomach, which induces early satiety and reduces caloric intake. By far, the most commonly used ballon in the United States, the Orbera^TM^ (Boston Scientific, El Coyol, Costa Rica) [13]. IGB is Food and Drug Administration (FDA) approved for a maximum duration of 6 months. During this timeframe, it can induce an excess weight loss of 30%–40% [14]. However, this weight loss is typically not durable, and weight often recurs once the balloon is removed. Given these limitations, ESG is the preferred endoscopic intervention for patients requiring more sustained weight loss and metabolic improvement. That said, for carefully selected patients who required rapid, short term weight reduction without permanent anatomical changes, IGB still remains a viable option.

Although longer-term follow-up is needed, ESG may be considered as an endobariatric approach in the AWR setting. ESG involves transoral placement of full thickness sutures to reduce the stomach’s size by 70%–80%, mimicking the restrictive anatomy of a surgical sleeve gastrectomy that limits food intake and induces changes in gastrointestinal hormones involved in satiety without the need for resection [15]. Importantly, this technique avoids entering the peritoneal cavity, which is particularly advantageous in patients with prior abdominal surgeries or mesh implantation. ESG produces most of the weight loss within 6 months, which is beneficial for a patient who is being optimized for a hernia repair. In the multi-center ESG Randomized Interventional Trial (MERIT-Trial), investigators enrolled 209 participants with class 1 or class 2 obesity (BMI 30–40 kg/m^2^) to assess the effects of ESG combined with lifestyle modifications [15]. After 52 weeks, the primary endpoint of 25% excess weight loss (EWL) was met in 77% of patients in the ESG group compared to only 12% in the control group (lifestyle modifications). Put differently, the mean percentage of total body weight loss at 52 weeks was 13.6% for the ESG group and 0.8% for the control group. Impressively, 68% of patients undergoing ESG maintained their weight loss at 2 years. ESG was well tolerated, with only 2% of participants experiencing serious adverse events (e.g., severe nausea, vomiting, and abdominal pain), none of which were life-threatening. The overall rate of major complications from sleeve gastrectomy is approximately 2%–7% (e.g., staple line leak, bleeding stricture or stenosis, and gastroesophageal reflux). Recent systematic review and meta-analysis yielded similar results with a 16.2% TWL at 12 months [16]. These results reinforce ESG as an effective and safe intervention for achieving sustainable weight loss in patients with obesity when compared to lifestyle modifications.

Beyond weight loss, ESG offers clinically significant benefits in metabolic health. In the MERIT trial, over 90% of the patients experienced clinical improvement in their diabetes, with no deterioration. A separate systematic review by Nunes et al. demonstrated that ESG is associated with significant improvement in hepatic steatosis, liver fibrosis, anthropometric measurements, and a reduction in HbA1c over 12 months of follow-up [17]. Such systemic improvements enhance surgical candidacy and long-term health outcomes, making ESG a compelling tool in multidisciplinary, prehabilitation protocols.

## Glucagon-Like-Peptide-1 (GLP-1) Agonist

The emerging data on GLP-1s in the context of AWR are also playing an increasingly prominent role in AWR prehabilitation. Their key benefit is immediacy; patients can initiate therapy promptly and begin losing weight without procedural clearance delays. The use of GLP-1s has demonstrated accelerated weight loss compared to lifestyle modifications alone and compress the timeframe to surgery [18, 19]. In a recently published prospective, single-institution hernia database analysis, patients who were optimized through diet and exercise alone took an average of 10 months to get to surgery while patients on GLP-1s have reached optimization targets at just over 6 months [10, 18]. Although there is no level 1 data directly comparing the weight loss effects of ESG and GLP-1s, GLP-1s may be particularly useful for patients who are not candidates for endoscopic procedures and require rapid preoperative optimization. This recommendation reflects considerations beyond weight loss alone, including procedural access, patient preferences, and the relative invasiveness of ESG compared with pharmacologic therapy.

GLP-1s do, however, have limitations. Medication tolerance is a significant concern; over one-third of patients experience significant gastrointestinal side effects, such as nausea and vomiting [20]. It should be noted that similar gastrointestinal symptoms (e.g., nausea and vomiting) have been reported following ESG; however, symptoms are typically limited to the immediate post-procedural period, as demonstrated in the MERIT trial and confirmed in systematic review [21]. Additional contraindications to GLP-1 therapy include a history of pancreatitis, and, in some instances, chronic kidney disease [22]. Furthermore, the need for ongoing administration of the medication to sustain benefits presents a practical and financial challenge. When not covered by insurance, the out-of-pocket cost for GLP-1 medications in the U.S. frequently exceeds $1,000 per month, making long-term use financially burdensome [23]. However, the use of GLP-1s may serve as a complimentary treatment to ESG and other lifestyle modifications.

## Proposed Algorithm

**FIGURE 1:**
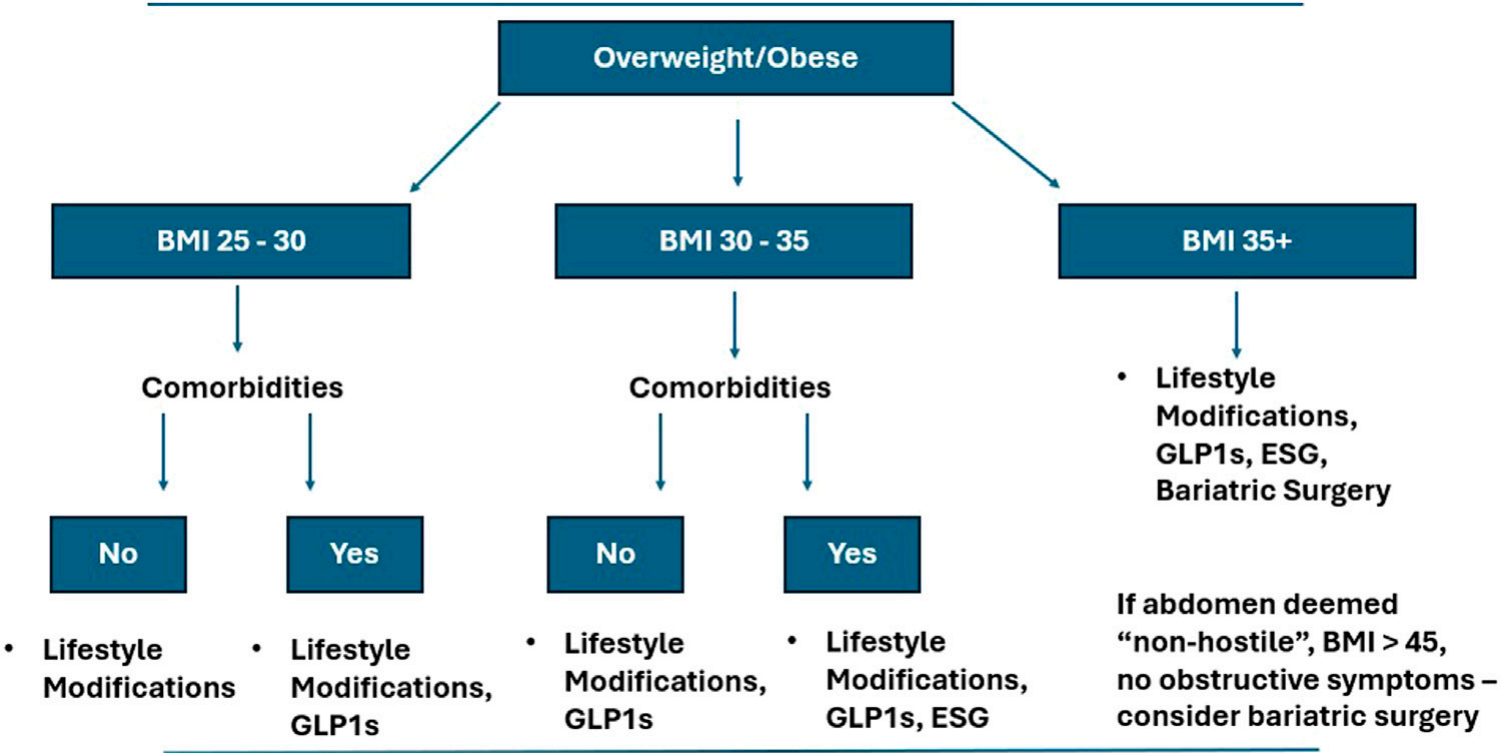
Algorithm for addressing obesity in abdominal wall reconstruction patients. The figure demonstrates a proposed algorithm for optimizing obese patients prior to abdominal wall reconstruction. The algorithm accounts for body mass index, comorbidities, and prior surgical history.

Figure 1 offers a practical algorithm for optimizing obese AWR patients prior to elective surgery. For all overweight patients and those with comorbidities (e.g., diabetes, NAFLD), initiate lifestyle modifications (ketogenic diet, aerobic exercise) and assess surgical urgency. If weight loss plateaus or urgency is low, consider ESG for BMI above 30 kg/m^2^ and ESG or bariatric surgery for patients with a BMI above 35 kg/m^2^ and with comorbidities. ESG would be the preferred treatment option in a patient with multiple prior abdominal surgeries. IGB would generally not be recommended as a first line procedural intervention given its lack of durability. GLP-1 agonists may be beneficial for more urgent cases or ESG-ineligible patients (e.g., prior gastric surgery). Concurrently, surgeons can address smoking cessation and glycemic control. For patients who have had recurrent episodes of incarceration, they may not be able to be optimized and require surgery prior to weight loss interventions. This algorithm requires validation through multicenter trials to optimize patient selection and outcomes. American and European Hernia Society-led initiatives should standardize endoscopic training to facilitate adoption as *part* of the algorithm to optimize obese patients. As of now, the defined optimization standards for minimally invasive abdominal wall reconstruction are less clear and may affect this algorithm.

## Discussion

ESG offers several unique advantages over traditional bariatric surgery in the setting of AWR. Unlike LSG or Roux-en-Y gastric bypass (LRYGB), ESG is incisionless and avoids intra-abdominal entry, which is important in patients with a hostile abdomen, prior mesh placement, etc. It is also associated with a shorter recovery time and reduced hospital stay, with most patients being discharged the same day and returning to normal activities shortly thereafter [15]. While LSG may provide greater weight loss than ESG in the long term, ESG demonstrates comparable improvement in comorbidities and superior procedural safety in the prehabilitation context [24]. LRYGB is less frequently performed in this setting because a hernia-related issue may lead to significant problems with intra-abdominal surgery, bariatric-related anatomy, and the possibility of future RYGB related surgery. ESG is particularly suited for patients with elevated BMI and diminished abdominal wall compliance, in whom even modest weight reduction can improve closure dynamics and reduce operative tension [25]. Additionally, ESG facilitates meaningful improvement in metabolic parameters, particularly diabetes and hepatic steatosis, thereby further reducing perioperative risk. As a same-day procedure with rapid recovery, ESG minimizes delays in care and can be initiated during the preoperative workup without major disruptions to the hernia surgical timeline. As endoscopic approaches to obesity management, particularly ESG, become mainstream, they have the potential to become an important part in the prehabilitation of patients undergoing complex hernia repair. While weight loss is only one component of comprehensive optimization (e.g., physical therapy, smoking cessation, glycemic control), surgeons who understand and utilize endoscopic options will be better positioned to offer individualized, effective care. Expanding the number of surgeons trained in endoscopic techniques has the ability to empower them to deliver integrated, in-house solutions that improve both access and outcomes. The successful integration of endobariatrics into clinical practice will ultimately depend on addressing issues such as insurance coverage, specialized training, and thoughtful patient selection. At present, ESG is not always covered by major commercialized health plans, but this is becoming less common as there is mounting evidence regarding its efficacy [26]. For these patients, bariatric surgery remains the gold standard procedural means for prehabilitation. The goal of this algorithm is to describe the role of endoscopic therapy as a potential option rather than assert its superiority to bariatric surgery, which is not the case.

Despite the promise that ESG may offer in the context of preoptimization, access remains a barrier. Many insurers do not deem ESG medically necessary, limiting access to patents who cannot pay out-of-pocket [27]. Although the defined and standardized code for reimbursement for ESG is forthcoming, its adoption into routine practice may be slowed by a lack of formally trained endoscopists. ESG requires specialized and advanced endoscopic skills not typically included in general surgery or gastroenterology training and often require specialized gastrointestinal or minimally invasive surgery fellowships or society-sponsored courses. These challenges may inevitably contribute to a slower uptake of ESG in hernia prehabilitation, despite its clear potential to improve patient outcomes. Surgeons who do offer ESG will be posed to uniquely take care of patients in the preoperative setting. As the aim of elective AWR is to improve patient quality of life, providing patients with the chance to have the optimal outcome and improve their overall health is paramount.
